# Mental Health Service Utilization among Black Youth; Psychosocial Determinants in a National Sample

**DOI:** 10.3390/children4050040

**Published:** 2017-05-17

**Authors:** Shervin Assari, Cleopatra Howard Caldwell

**Affiliations:** 1 Department of Psychiatry, School of Medicine, University of Michigan, Ann Arbor, MI 48109-2029, USA; 2 Center for Research on Ethnicity, Culture and Health, School of Public Health, University of Michigan, Ann Arbor, MI 48109-2029, USA; 3 Department of Health Behavior and Health Education, School of Public Health, University of Michigan, Ann Arbor, MI 48109-2029, USA; cleoc@umich.edu

**Keywords:** African Americans, Blacks, youth, gender, ethnicity, psychiatric disorders, mental health care need

## Abstract

Racial disparity in mental health service utilization (MHSU) persists, and youths are not an exception to the underutilization of services. Very limited research has been conducted on the determinants of MHSU among Black youth. Using a national sample of American Black youth, the current study investigated the association between demographic factors, socioeconomic status, psychiatric disorders, and self-rated health (SRH) on MHSU. We also tested the heterogeneity of the effects of SRH and psychiatric disorders based on ethnicity, gender, and their intersection. We used data from the National Survey of American Life-Adolescents supplement (NSAL-A), 2003–2004. The study enrolled 1170 Black youth between 13 and 17 years old including 810 African Americans and 360 Caribbean Blacks. Age, gender, ethnicity, socioeconomic status, SRH, 12-month psychiatric disorders (Composite International Diagnostic Interview modified version), and MHSU (last year) were measured. Logistic regressions were used for data analysis. Ethnicity (odds ratio (OR) = 0.33, 95% confidence interval (CI) = 0.17–0.65), subjective socioeconomic status (OR = 1.43, 95% CI = 1.09–1.88), SRH (OR = 2.45, 95% CI = 1.00–6.37), and psychiatric disorders (OR = 2.17, 95% CI = 1.05–4.48) were associated with MHSU. Age, gender, and objective socioeconomic status were not associated with MHSU. Gender and ethnicity did not interact with SRH and psychiatric disorders on MHSU. Actual and perceived need both universally influence Black youths’ likelihood of MHSU, regardless of their ethnicity and gender. Ethnicity and perceived socioeconomic status also play unique roles in MHSU. Future research is needed to understand pathways to MHSU for Black youth who both have and perceive mental health needs. There is also a need to find ways to promote MHSU for those with a need for mental health services.

## 1. Background

Youth are not an exception to the persistent racial disparities in mental health service utilization (MHSU) [1]. Similar to Black adults [2,3,4,5], Black youth are more likely to underutilize mental health services than Whites [6,7]. This is a public health concern as undiagnosed and untreated psychiatric disorders increase the risk of a wide range of negative outcomes including poor upward social mobility and reduced social capital in adulthood [8]. Untreated youth depression, for example, increases future welfare dependence and unemployment among adults [8].

Finding solutions that can contribute to the elimination of racial and ethnic disparities in MHSU requires knowledge on barriers and facilitators of MHSU among Black youth. While systemic and societal barriers are also important, individual psychosocial determinants of MHSU also have a role [9]. Individual psychosocial factors may or may not vary across different ethnic groups of Black youth. Previous research is very limited in this regard.

Low socioeconomic status and perceived financial difficulty increase the risk of serious psychiatric disorders in youth [10]. Socioeconomic disadvantage specifically heightens the risk of depression, anxiety, conduct disorder, and substance use among minority youth [11,12]. Low socioeconomic status may also affect MHSU in individuals with an actual need for mental health services [13,14]. While socioeconomics factors explain some of the Black–White disparities in MHSU [5,15], they also influence patterns of MHSU within Blacks [4,5].

The self-rated mental health (SRH) measures evaluate both actual [16,17,18,19,20] and perceived [21,22] need for health care. The single-item SRH measure is associated with psychological distress [23] and a wide range of psychiatric disorders such as major depressive disorder (MDD) and generalized anxiety disorder (GAD) [15,19,20,24]. They also correlate with psychiatric disorders [16,25], help-seeking behaviors [21] and adherence to psychiatric prescriptions [22]. Poor SRH may prompt complex cognitive processes that are involved in MHSU [9,26,27]. Help seeking is very unlikely unless the individual perceives his or her own health as poor [21,25,26,27,28,29,30,31,32,33,34].

Even within a particular racial group, ethnicity influences distribution of need, preferences, and use of mental health care [35]. While both are considered Black, African Americans and Caribbean Blacks differ in mental health needs [36], utilization of professional care [35,37,38,39,40], and the impact of actual need on their perceived need [41]. In a recent study, MDD and GAD were found to have affected SRH of Caribbean Blacks and African Americans, respectively [41].

Using a national sample of ethnically diverse Black youth, the current study investigated the effects of demographic factors, socioeconomic status, psychiatric disorders, and SRH on MHSU. We also tested the gender and ethnic heterogeneity of the effects of SRH and psychiatric disorders on MHSU in our sample.

## 2. Materials and Methods

### 2.1. Design and Setting

This cross-sectional study used data from the National Survey of American Life-Adolescents (NSAL-A) supplement, 2003 [42,43]. The NSAL-A is one of the largest national mental health surveys of an ethnically diverse sample of Black youth ever conducted in the United States. As a part of the Collaborative Psychiatric Epidemiology Surveys (CPES), the study was funded by the National Institute of Mental Health (NIMH) [42,43,44].

### 2.2. Ethical Considerations

The University of Michigan (UM) Institute Review Board (IRB) approved the NSAL study (ID code H03-00001309-R2). Youths’ legal guardians provided informed consent. Assent was obtained from the youth. Each individual received $50 as financial compensation. Data were kept fully confidential.

### 2.3. Participants and Sampling

The NSAL-A included a total of 1170 Black youth. This number was comprised of 810 African American and 360 Caribbean Blacks adolescents. All adolescents were between 13 and 17 years old. Youth between 13 and 17 years old were selected as they are more homogenous and similar developmentally compared to adolescents of a wider age range (e.g., 13 to 21 years old).

While African Americans were sampled from large cities or other urban and rural areas, the Caribbean Black sample was exclusively selected from large cities. The NSAL used a national household probability sample of Blacks. All African American and Caribbean Black households included in the NSAL-Adult survey were screened for at least one eligible youth living in the household. Youth were then randomly selected from the provided list. In the presence of multiple eligible youth in the household, two adolescents were selected based on the gender of the first selected adolescent. Thus, the NSAL-A sample was non-independent. As a result, the NSAL-A data were weighted to adjust for non-independent selection probabilities within the households, clustering, strata, and non-response across households and individuals. At the final step, the NSAL-A weighted data were post-stratified to represent the national estimates of Black youth based on gender and age [45,46]. More details on the NSAL sampling are available elsewhere [43,44].

### 2.4. Data Collection

About 82% of the data was collected in face-to-face home interviews. The remaining 18% of the interviews were either entirely or partially conducted through telephone. Computer-assisted personal interviews (CAPIs) were used to enhance the quality of the face-to face interviews. CAPI is a preferred interviewing technique particularly for long and complex survey tools [47]. All interviews were performed in the English language and lasted 100 min on average. The overall response rate was 81%, with 80% for African Americans and 83% for Caribbean Blacks.

### 2.5. Survey Measures

*Demographic factors.* The major demographic factors measured were age and gender. Age was measured as a continuous variable and male was the referent category for gender, which was a dichotomous variable.

*Socioeconomic status.* Objective and subjective measures of socioeconomic status were both measured in this study. For objective socioeconomic status, we calculated the income-to-needs ratio as an indicator for poverty status based on a measure of family income and number of individuals in the household. The study also measured perceived economic hardship using a single item asking adolescents how they perceived their family of origin’s financial situation. The question read, “In general, would you say you and your family living here have more money than you need, just enough for your needs, or not enough to meet your needs?” Responses included (1) more than need, (2) just enough, and (3) not enough. We combined the above three responses to create a dichotomous variable “not enough money” versus “just enough or more than needed money”. Other researchers have used subjective socioeconomic status and perceived financial hardship [48,49,50].

*Self-Rated Health* (*SRH*). Participants were asked “How would you rate your overall physical health?” Responses included five categories including excellent, very good, good, fair, and poor. We collapsed responses into two categories, poor/fair and excellent/very good/good [51,52]. Single-item SRH measures are associated with several aspects of health and well-being [53] and have shown high test–retest reliability, ranging from 0.7 to 0.8 for brief time intervals [54]. These single items strongly correlate with longer scales [54]. SRH measures also predict risk of mortality above and beyond traditional risk factors [55].

*Race* versus *Ethnicity.* This study is based on within-race (i.e., Black) ethnic (e.g., African Americans or Caribbean Blacks) heterogeneities. Different from race, ethnicity refers to people based on their culture, beliefs, values, norms, geographic region, life history, etc. In this view, while both race and ethnicity share an ideology of common ancestry, they are distinct social constructs. Race and ethnicity are both self-identified by the individuals. While race is associated with social history as well as biology (e.g., genetic differences), ethnicity is more closely associated with cultural heritage and life history. Skin color, skin tone, facial features, and hair color and texture are cues of race but not ethnicity, however, traditions, life experiences, values, cultures, and religions are reflective of ethnicity.

*Ethnicity.* Youth ethnicity was measured according to the ethnicity of the parents who were living in the same household. Parents self-identified as African American if they were Black without any ancestral ties to the Caribbean. Parents self-identified as Caribbean Black if they were Black and had ancestral ties to one of the following Caribbean countries (*n* = 13): (1) Cuba, (2) Dominican Republic, (3) Haiti, (4) The Bahamas, (5) Jamaica, (6) Trinidad and Tobago, (7) Dominica, (8) Saint Lucia, (9) Antigua and Barbuda, (10) Barbados, (11) Saint Vincent and the Grenadines, (12) Grenada, and (13) Saint Kitts and Nevis.

*Lifetime Psychiatric Disorders.* Lifetime (non-psychotic) psychiatric disorders were measured based on the Diagnostic and Statistical Manual, 4th Edition (DSM-IV-TR) criteria. The modified Composite International Diagnostic Interview (CIDI) was utilized for this purpose [56,57]. Originally developed for the World Mental Health project in 2000 [56], the CIDI is a comprehensive fully structured interview schedule that is designed to be used by trained, lay interviewers. The CIDI is widely used in national and international epidemiological studies and has demonstrated high reliability and validity [57,58,59]. The prevalence of differences between the CIDI and the Structured Clinical Interview for DSM-IV-TR (SCID) has been shown to be insignificant [58]. CIDI is believed to provide valid findings for Blacks and their ethnic groups [60,61].

In this study, lifetime psychiatric disorders, was a dichotomous variable indicating presence of any of the following psychiatric disorders: mood disorders (i.e., major depressive disorder, dysthymia, bipolar I and II disorders), anxiety disorders (i.e., generalized anxiety disorder, panic disorder, agoraphobia, social phobia, separation anxiety disorder, obsessive–compulsive disorder, posttraumatic stress disorder), substance use disorders (i.e., alcohol abuse, alcohol dependence, drug abuse, drug dependence), conduct disorder, attention deficit/hyperactivity disorder, oppositional defiant disorder, and eating disorders.

*Mental Health Service Utilization.* Self-reported MHSU for emotional problems was the main outcome in this study. Data utilized in this study came from the questions asked from all the participating youth, regardless of whether they endorse criteria for a psychiatric disorder. Respondents were asked: “In the past year, have you been to a medical person, not counting your school nurse, for emotional problems?” The same method using self-reported data has been used elsewhere [4,5,62,63,64,65].

### 2.6. Statistical Analysis

To account for the complex design of the NSAL-A, Stata 13.0 (Stata Corp., College Station, TX, USA) was used. Taylor series approximation technique was applied to estimate complex design-based standard errors (SEs). All the percentages reported depict nationally representative figures.

For multivariable analysis, we used survey logistic regressions with MHSU as the main outcome, demographics, socioeconomic status, SRH, and psychiatric disorders as the independent variables, and ethnicity and gender as moderators. In order to determine the moderating role of gender and ethnicity, we ran a model with the main effects only, followed by models with interactions between gender/ethnicity with SRH, and psychiatric disorders. Adjusted odds ratio (OR), SE, 95% confidence interval, and *p*-value were reported. A *p*-value of less than 0.05 was considered statistically significant.

## 3. Results

Participants were 15 years old (standard deviation (SD) = 1.42) on average and had the following age distribution: 13–14 (*n* = 477, 40%), 15–16 (*n* = 441, 41%) and 17 (*n* = 252, 19%) years old. There were slightly more girls (*n* = 605, 52%) than boys (*n* = 563, 48%). Of the 1170 youth, 96% identified as a high school student. Family income ranged from $0 to $520,000, with a median of $28,000. Caribbean Blacks had a higher median income ($32,250) compared to African Americans ($26,000) (*p* < 0.001). Lifetime psychiatric disorder was more common among Caribbean Blacks than among African Americans. With a marginally significant association, lifetime psychiatric disorder was more common among boys than girls (*p* = 0.1).

Table 1 describes distribution of gender, ethnicity, and age in the pooled sample, and based on ethnicity and gender.

Table 2 summarizes our logistic regression in the pooled sample. Caribbean Black Ethnicity was associated with lower MHSU (OR = 0.33, 95% confidence interval (CI) = 0.17–0.65), while worse subjective socioeconomic status (OR = 1.43, 95% CI = 1.09–1.88), poor SRH (OR = 2.45, 95% CI = 1.00–6.37) and endorsing DSM-IV criteria for at least one psychiatric disorder (OR = 2.17, 95% CI = 1.05–4.48) were associated with higher odds of MHSU. Age, gender, and objective socioeconomic status were not associated with MHSU. In addition, gender or ethnicity did not interact with SRH or psychiatric disorders on MHSU.

## 4. Discussion

These findings suggest that ethnicity, subjective socioeconomic status, psychiatric disorders, and SRH all determine MHSU among Black youth.

In our study, Black youth’s MHSU was shaped by proxies of their culture/values, subjective financial hardship/barriers, actual need, and perceived need. These findings can be explained by the Andersen healthcare utilization model [66,67]. According to this model, utilization of health services (including MHSU) is determined by predisposing factors, enabling factors, and need factors [66,68]. Need is composed of both perceived and actual need for health care services. Ethnicity influences MHSU through culture, norms, and values, which shape mistrust and stigma. All these factors can be conceptualized as predisposing characteristics. Finally, socioeconomic status can be seen under enabling factors [69,70].

In this study, ethnicity affected MHSU, net of socioeconomic status, actual need, and perceived need. Previous research has shown that ethnicity alters MHSU among Black adults [5]. Our findings also suggest that ethnicity should be considered in the efforts to promote MHSU of Black youths. Ethnic-specific strategies may also help with promotion of help seeking behaviors as ethnic groups differ in stigma and mistrust, as well as values, culture, socioeconomic status, and discriminatory experiences, all of which have implications for MHSU [36,40,61,71,72]. Trust toward the health care system and stigma widely differ across ethnic groups, as they are shaped by historical life experiences. In the case of African Americans, there is a historical mistrust that may not exist for Caribbean Blacks [73,74,75,76,77,78]. The two groups also differ vastly in traditions [79,80]. In addition, socioeconomic status, life conditions under poverty and unsafe neighborhoods, history of systemic racism and discrimination, racial identity, and race consciousness are all specific to each ethnicity of Blacks [81,82,83].

In our study, ethnicity was associated with MHSU of Black youth, after adjusting for socioeconomic status, actual need (psychiatric disorder), and perceived need. Even when ethnic health differences arise through differences in socioeconomic status, insurance, or other mechanisms, the Institute of Medicine (IOM) defined them as unfair ethnic disparities [84]. Additional efforts should be made to within-race ethnic disparities in MHSU among Black families.

Subjective but not objective socioeconomic status was a determinant of MHSU in this study. This finding emphasizes that financial hardship may be a determinant of mental health care need. Based on the literature, poor socioeconomics and economic hardship both increase the risk of psychiatric disorders such as depression, anxiety, conduct disorder, and substance abuse among minority youth [10,11,12]. In addition to mistrust and stigma, low socioeconomic status has been listed as one of the reasons minority individuals including Blacks underutilize health care [5,13,14,15]. Socioeconomic status is both relevant to within- and between-ethnic variation in MHSU among African Americans and Caribbean Blacks [4,5]. Future research should investigate what proportion of the effect of family socioeconomic status on MHSU is due to stigma, mistrust, low awareness, and poor access.

Psychiatric disorders and SRH both had additive effects and neither fully accounted for the MHSU of Black youth. This finding suggests that SRH is a useful tool to detect individuals in need for mental health care. The study did not show ethnic or gender differences in the effects of actual or perceived need on MHSU. In other words, in the presence of need (poor SRH or psychiatric disorders), ethnic and gender groups would similarly seek mental health care. These findings suggest that need has a universal impact on MHSU of gender and ethnic groups among Black youth.

Very little is known about the role of SRH in linking actual need to service use among Black youth [25]. According to the literature, the meaning of SRH varies across ethnic groups as SRH may reflect different health needs across demographic and social groups [18,85,86]. Overall, among adults, poor SRH better reflects mental disorders in Whites compared to Blacks, Hispanics, and Asians [18]. Even within a single racial group, SRH differently correlates with psychiatric disorders across ethnic groups [86]. We, however, did not find evidence regarding an interaction between ethnicity and SRH on MHSU in our sample of Black youth.

Our findings suggest that SRH is a useful screening tool for detection of mental health care need across both ethnic groups of Black youth. In a previous study among adults, SRH better indicated risk of GAD and MDD for adult African Americans and Caribbean Blacks, respectively [86]. Despite previous findings that SRH may differently correlate with psychiatric disorders across ethnic groups of adults [85,87,88,89], SRH was universally linked to MHSU across all ethnic and gender groups of Black youth. In the Medical Expenditure Panel Survey (MEPS) 2000–2004, the effect of SRH on service use was moderated by ethnicity [30]. Most of the evidence related to differential meaning of SRH is across racial but not ethnic groups [88,89,90].

This study provides additional information regarding the process of decision-making to seek specialty mental health care [91]. Although it is not very clear what single item SRH represents across minority groups [18,24,85,86,92,93], SRH is one of the determinants of mental health care use among Black youth. This is partially due to the fact that SRH reflects psychological distress, depressive symptoms [24], as well as psychiatric disorders [18,86].

Our findings may have implications for the promotion of MHSU. Among those with a psychiatric disorder, there are ethnic groups who generally do not seek professional care, and require additional policies and programs to support them in this regard.

The current study had a few limitations. First, the sample size was not balanced across all subpopulations. This problem may result in differential statistical power across race by gender groups. Second, some of the study constructs may have differential validity across our subpopulations. Future research should compare ethnic groups of Black youth for validity and reliability of MHSU. Third, knowledge about appropriate local health services and the pathways to accessing them were not measured in this study. Health service utilization is strongly influenced by knowledge about local mental health services and knowledge about how to access such services. Future research should investigate their potential influence on MHSU of Black families. Finally, we could not break the results based on states. This study used publicly available data which do not include information on states.

As the data reported here were collected in 2003, there is a need to replicate these findings using more updated surveys. Societal and political context in the United States has changed enormously since 2003, and major changes have changed to the life circumstances of minorities including Blacks. One example is the changes in the health care reform first in Obama administration and then in the Trump administration. Another considerable change is an increase in the accessibility and popularity of internet resources and social media among adolescents. At the same time, patterns of particular types of mental disorders such as substance use and abuse are subject to change, secondary to the emergence of new legal and illicit drugs. Despite these limitations, given that the existing knowledge is so limited, we believe that these findings extend the existing knowledge on the MHSU determinants.

Although data were collected by various survey modes, this is common practice. Many national surveys use a combination of various data collection modes. As variables in this study were not sensitive, they are unlikely to be affected by the mode of interview. Data collection mode was also not a part of publicly available data which was used for this study. In addition, immigration status and nativity were not a threat to the validity of this study. Only a handful of African American and Caribbean Black youth were not U.S.-born.

There is still a need for future research on the complex relationships among race, ethnicity, objective socioeconomic status, perceived financial hardship, psychiatric disorders, SRH, and MHSU of minority youth [18]. Findings from such studies would contribute to eliminating or at least reducing ethnic disparities in MHSU. Although not supported by this paper, these associations may not be linear and additive, as ethnicity, gender, and socioeconomic status may change the effects and meanings of SRH and psychiatric disorders on the life of the individuals [86].

To conclude, ethnicity, subjective socioeconomic status, psychiatric disorders, and SRH all determine MHSU among Black youth. The effects of actual and perceived need (i.e., psychiatric disorders and SRH) on MHSU were universal across gender and ethnic groups of Black youth. The findings help us better understand the process and determinants of MHSU among Black youth.

## Figures and Tables

**Table 1:** Descriptive statistics in the pooled sample and based on ethnicity and gender.

					Gender								Ethnicity							
	All				Males				Females				African Americans (AA)				Caribbean Blacks (CB)			
	%	SE	95% CI		%	SE	95% CI		%	SE	95% CI		%	SE	95% CI		%	SE	95% CI	
Gender																				
Male	50.02	0.02	46.49	53.54									50.39	0.02	46.57	54.20	44.78	0.02	39.98	49.68
Female	49.98	0.02	46.46	53.51									49.61	0.02	45.80	53.43	55.22	0.02	50.32	60.02
Ethnicity																				
AA	93.37	0.01	91.89	94.60	94.07	0.01	92.69	95.20	92.68	0.01	90.64	0.94.31								
CB	6.63	0.01	5.40	8.11	5.93	0.01	4.80	7.31	7.32	0.01	5.69	0.09.36								
Age	14.98	0.06	14.86	15.10	14.98	0.07	14.84	15.12	14.98	0.09	14.80	15.15	14.96	0.07	14.83	15.10	15.22	0.06	15.09	15.35

CI: confidence interval; SE: standard error.

**Table 2:** Logistic regression in the pooled sample.

	OR (SE)	95% CI	*p*-Value
**Demographics**			
Ethnicity	0.33 (0.11)	0.17–0.65	0.002
Gender (Females)	1.23 (0.31)	0.74–2.03	0.411
Age	0.96 (0.14)	0.72–1.28	0.770
**Socioeconomics**			
Poverty Index	0.94 (0.08)	0.78–1.12	0.458
Subjective Socioeconomic Status	1.43 (0.19)	1.09–1.88	0.010
**Mental Health**			
Any Psychiatric Disorders	2.17 (0.78)	1.05–4.48	0.037
Poor Self-Rated Health	2.45 (1.16)	1.00–6.37	0.050
**Intercept**	0.04 (0.08)	0.00–3.78	0.157

OR: odds ratio.
